# A Cross-Sectional Study of Congenital Adrenal Hyperplasia

**DOI:** 10.7759/cureus.63520

**Published:** 2024-06-30

**Authors:** Ilham Bouarab, Fatimazahra Yakine, Bouchra Slaoui

**Affiliations:** 1 Pediatrics Department, Abderrahim Harouchi Mother-Child Hospital, Ibn Rochd University Hospital, Casablanca, MAR

**Keywords:** 17-hydroxyprogesterone, 21-hydroxylase, congenital adrenal hyperplasia, disorders of sexual development, salt-wasting syndrome

## Abstract

Introduction and aim: Congenital adrenal hyperplasia is an autosomal recessive disease caused by the deficiency of one of the enzymes of adrenal steroidogenesis, the most common of which is the deficiency of 21-hydroxylases. It represents a significant cause of morbidity and mortality in the pediatric population, especially in the absence of systematic neonatal screening in Morocco, which makes the management of these patients difficult for clinicians. This study aimed to describe the epidemiological, clinical, laboratory, evolutionary, and therapeutic profile of children followed for congenital adrenal hyperplasia at the pediatric endocrinology unit, Abderrahim Harrouchi Children's Hospital, Casablanca, Morocco.

Materials and methods: A retrospective cross-sectional study including 184 children followed for congenital adrenal hyperplasia over a period of 11 years (from January 1, 2013, to December 31, 2023). The diagnosis was confirmed by molecular biology, and all clinical, laboratory, and radiological data were collected retrospectively from medical records.

Results: The median age at diagnosis was 1.5 months (birth: 13 years). The consanguinity rate was 54.4% (n=100). A history of death in the family was found in 16.3% (n=30) of cases in a table of salt wasting and infections. The classic form was observed in 72% (n=132) of children compared to 28.3% (n=52) for the non-classical form. The virilizing form with salt wasting and the pure virilizing form represented 45.6% (n=84) and 26% (n=48) of cases, respectively. Deficiency in 21-hydroxylase was found in 91.8% (n=169) of children, while deficiency in 11-β-hydroxylase was identified in 4.9% (n=9) of cases, and in 3-β-hydroxysteroid dehydrogenase in 3.2% (n=6) of cases. A total of 40.7% (n=75) of children underwent corrective surgery of the external genitalia.

Conclusion: Congenital adrenal hyperplasia is a group of rare diseases. The best therapeutic alternative would be newborn screening and antenatal diagnosis.

## Introduction

Congenital adrenal hyperplasia (CAH) is an autosomal recessive disease that occurs due to a group of enzymatic deficiencies in the adrenal steroidogenesis pathway. Its worldwide incidence varies in most studies between 1 in 15000 and 1 in 16000 births [1,2]. Depending on the type and severity of the enzymatic block, various alterations in the production of glucocorticoids, mineralocorticoids, and sex steroids can occur [2].

Deficiency in 21-hydroxylase is the most common form (90-95%), associated with mutations in the CYP21A2 gene. This enzyme converts 17-hydroxyprogesterone (17-OHP) into 11-deoxycortisol and progesterone into deoxycorticosterone, these products being precursors of cortisol and aldosterone. Patients with this deficiency exhibit various phenotypes due to residual broad-spectrum enzymatic activity of different CYP21A2 mutations [1].

Severe forms, known as classical, are characterized on the one hand by cortisol deficiency and sometimes aldosterone deficiency, which can be life-threatening, and on the other hand by an increase in the synthesis of adrenal androgens leading to virilization of external female genitalia, ranging from mild clitoral hypertrophy to a male phenotype with a penis and scrotal bursae. The non-classical form manifests itself later by peripheral or central precocious puberty, advanced stature, and signs of hyperandrogenism (hirsutism, acne), or it may remain asymptomatic and reveal itself in adulthood with signs of infertility [1,2].

Numerous scientific studies have proven that early diagnosis of CAH and, therefore, early intervention would enable the implementation of appropriate care and advice aimed at minimizing long-term complications and comorbidities [2]. Unfortunately, systematic screening through the measurement of 17-hydroxyprogesterone (on blotting paper) has not yet been carried out in Morocco, the aim of which is to reduce morbi-mortality associated with salt-wasting syndrome, prevent gender misassignment in virilized girls, and limit the effects of long-term hyperandrogenism.

To our knowledge, no large series of CAH cases have been reported in Morocco. The objective of this study was to describe the epidemiological, clinical, paraclinical, evolutionary, and therapeutic profile of children followed for CAH at the Pediatric Endocrinology Unit, Abderrahim Harrouchi Children's Hospital, Casablanca, Morocco. To achieve this objective, we analyzed the characteristics and the biological and molecular profiles of 184 children suffering from CAH.

## Materials and methods

We conducted a cross-sectional, descriptive, retrospective study of newborns, infants, and children with congenital adrenal hyperplasia followed at the Pediatric Endocrinology Unit at the Abderrahim Harouchi Mother-Child Hospital in Casablanca. A total of 184 newborns, infants, and children diagnosed with congenital adrenal hyperplasia over 11 years between January 2013 and December 2023 were included in this study. The diagnosis was confirmed by molecular biology. Genetic studies were not conducted due to the unavailability of such services in our country.

In this study, we included all children under 15 years of age with a clinical diagnosis of CAH and 17-hydroxyprogesterone (17-OHP) levels >1000 ng/dL. We excluded incomplete records and all patients over 15 years of age. Medical records were reviewed and information was recorded in Excel to create a database. Only four newborns were screened at birth in the private sector because they had a family history of congenital adrenal hyperplasia. The other cases were diagnosed after presenting with symptoms related to congenital adrenal hyperplasia, as systematic screening at birth has not yet been carried out in our country.

All epidemiological, clinical, and biological data were collected retrospectively from patients' medical records. Regarding epidemiological characteristics, we were able to collect the initial age at diagnosis, sex assigned at birth, and urban or rural origin. We divided the children into the following three categories: those with the classic form with salt-wasting, those with the pure virilizing form, and those with the non-classic form. We also collected the clinical manifestations of the different phenotypes, i.e., sexual differentiation disorders classified according to the Prader score, salt-wasting syndrome, and signs of hyperandrogenism for the non-classical form, as well as the associated comorbidities, such as stature retardation, stature advancement, and precocious puberty. Chromosomal analysis was performed to confirm genotypic sex, while pelvic ultrasound was performed to assess internal sex organs.

We analyzed the different phenotypes according to the results of the biological tests, based on the 8-hour cortisol level, the 17-hydroxyprogesterone values, the karyotype, and the type of enzyme block, i.e., 21-hydroxylase, 11-beta-hydroxylase and 3-β-hydroxysteroid dehydrogenase block.

All the children who presented with a sexual differentiation disorder underwent a multidisciplinary consultation involving the pediatric endocrinologist, the pediatric surgeon, and the psychologist. Postoperative follow-up was also conducted by the respective specialists.

## Results

This cohort comprised 184 children whose congenital adrenal hyperplasia was confirmed by molecular biology. Retrospective analysis of medical records revealed that the ages of the children ranged from birth to 13 years, with a median age of 1.5 months at the time of diagnosis. Neonatal diagnosis was made in 33.2% (n=61) of cases. The consanguinity rate was 54.4% (n=100). A family history of similar cases was found in 46% (n=85) of cases, of which 35.3% (n=30) resulted in deaths due to salt wasting. Table 1 presents the epidemiological and sociodemographic characteristics of the children included in this study, including sex assigned at birth, age at diagnosis, and urban or rural origin.

**Table 1:** Sociodemographic characteristics of the study population.

Variables	n (%)
Gender assigned at birth	
Girls	88 (47.8)
Boys	89 (48.4)
Unassigned	7 (3.8)
Age at the time of diagnosis	
<30 days	61 (33.2)
1-12 months	70 (38)
>12 months	36 (19.5)
Not reported	17 (9.3)
Origin	
Urban	130 (70.6)
Rural	54 (29.4)

Disorders of sex development (DSD) were the most frequent mode of revelation in 26% of cases (n=48), followed by isolated salt-wasting syndrome in 25% (n=46) of cases, salt-wasting syndrome associated with DSD in 20.6% of cases (n=38), precocious puberty in 14% of children (n=26), and signs of hyperandrogenism in 14% of cases (n=26). Neonatal screening was performed in only 2.2% (n=4) of cases.

**Figure 1:**
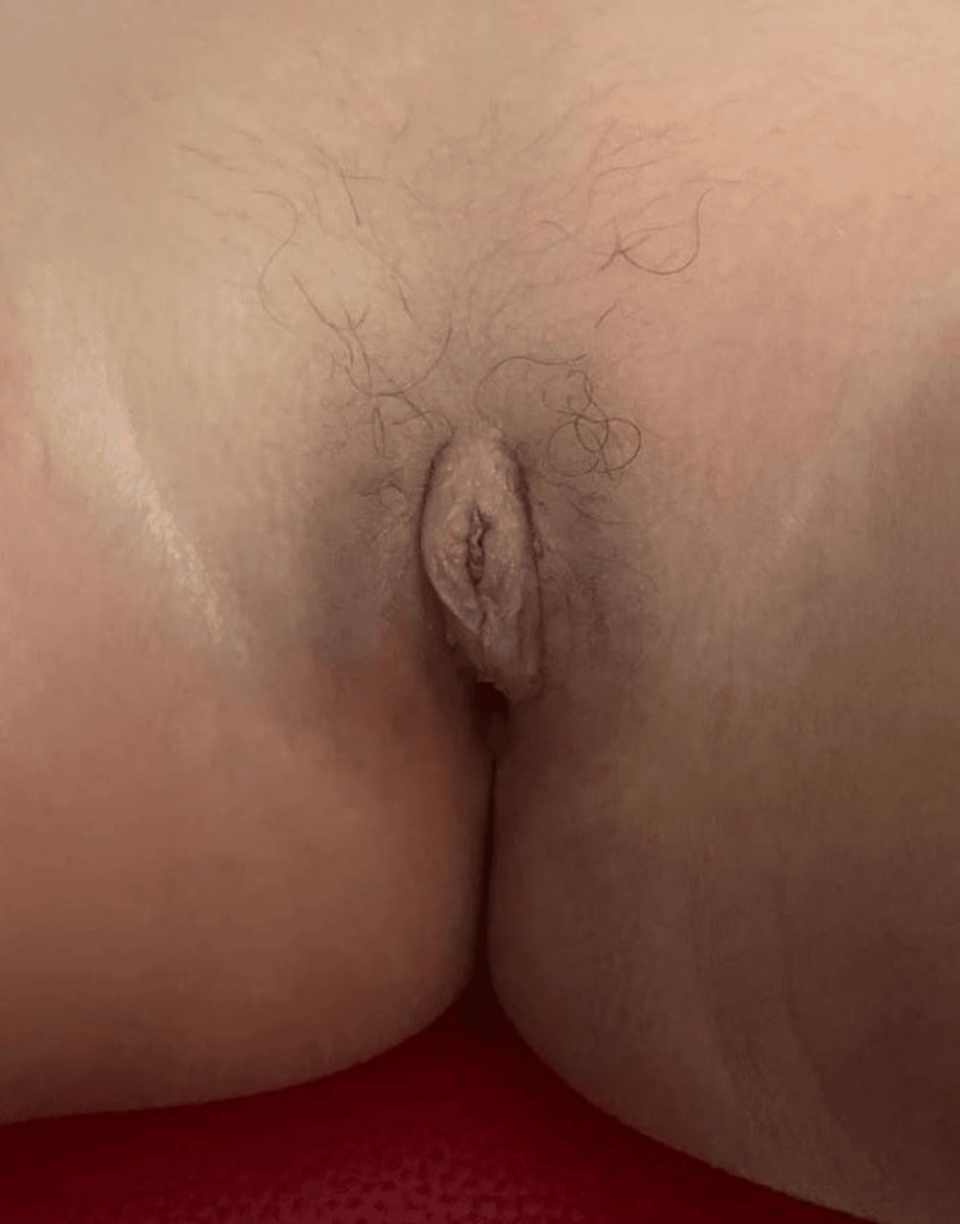
Clitoral hypertrophy with posterior labial fusion (stage II of Prader scale).

**Figure 2:**
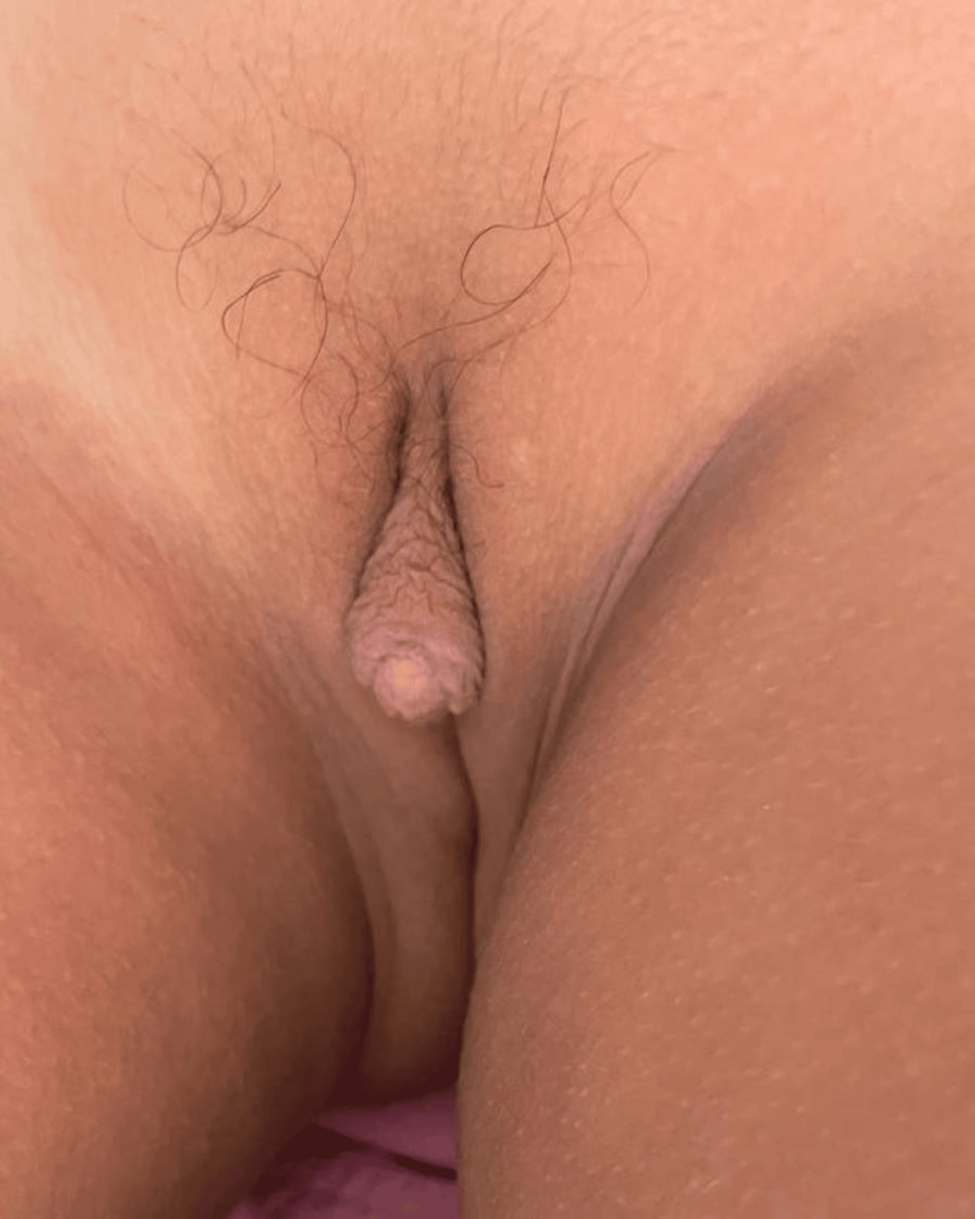
Higher degree of clitoral hypertrophy with a urogenital sinus and almost complete labial fusion (stage III of Prader scale).

**Figure 3:**
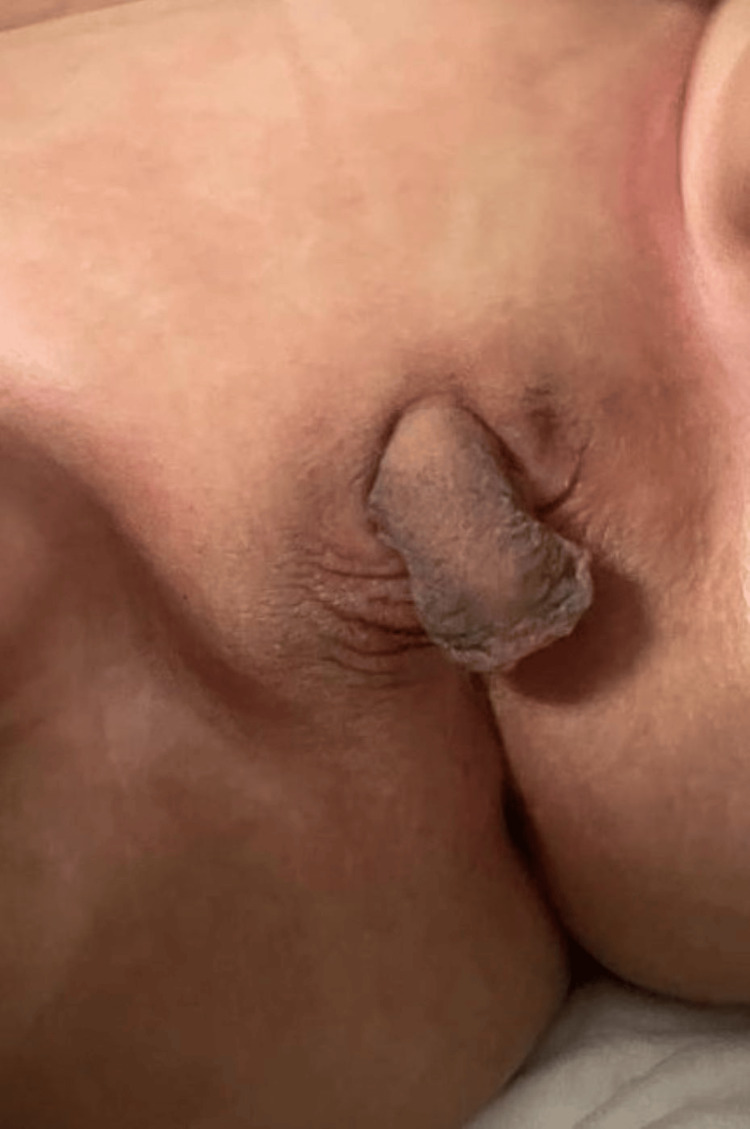
Penoclitoral organ, urethral meatus at the tip of the phallus, fused pseudo-scrotal genital folds, without palpated gonads (stage V Prader scale).

The classic form was the most frequent in this study, accounting for 71.7% (n=132) of cases, followed by the non-classic form in 28.3% (n=52) of cases, as shown in Table 2, which also summarizes the frequency of the different stages of the Prader score. The stages are also presented in Figures 1, 2, 3, showing images of children with disorders of sexual differentiation stages II, III, and V, respectively.

**Table 2:** Clinical characteristics of the study population.

Features	n=184
Clinical forms, n (%)	
Classical form with salt wasting	84 (45.6)
Pure virilizing form	48 (26.1)
Non-classic form	52 (28.3)
Prader score, n (%)	
Female	10 (5.4)
1	6 (3.2)
2	25 (13.6)
3	25 (13.6)
4	37 (20.1)
5	19 (10.3)
Male	39 (21.2)
Not reported	23 (12.5)
Associated comorbidities, n (%)	
Short stature	48 (26.1)
Stature advance	35 (19)
Precocious puberty	26 (14)
Obesity	8 (4.3)
Cushing syndrome	3 (2)
Arterial hypertension	2 (1)
Hypophosphatemic rickets	1 (0.5)

Biochemical diagnosis by 17-OHP assay was carried out in all patients before treatment initiation, and it was elevated compared to the corresponding normal value for age in 98.9% (n=182) of cases, with a median of 5560 ng/dL (range: 1190-30080 ng/dL).

Salt-wasting syndrome was observed in 45.6% (n=84) of cases. Electrolyte disorders such as hyponatremia and hyperkalemia were observed in 84.5% (n=71) of cases, with a median serum sodium level of 125 mEq/L and a median serum potassium level of 6.5 mEq/L. Isolated hyponatremia was present in 15.4% (n=13) of cases. Plasma renin was elevated in 57.2% (n=48) of cases, with a median of 3200 pg/mL, associated with hypoaldosteronism. Cortisolemia was low in 70.6% (n=130) of cases with a median of 4.5 µg/dL (range: 2.3-6.7), and high adrenocorticotropic hormone (ACTH) with a median of 60 pg/mL (range: 13-104 pg/mL). Testosterone assay was high in 86% (n=158) of cases.

A pelvic ultrasound was performed in all cases, revealing female internal genital organs in 45.1% (n=83) of cases, of which 38.5% (n=32) were newborns declared male at birth. A blood karyotype was requested in all children but was carried out in only 77.7% (n=143) of cases, with 74.8% (n=107) having a 46XX karyotype and 24.5% (n=35) having a 46XY karyotype. One case had a 45X karyotype related to the associated Turner syndrome.

Regarding the etiological diagnosis, molecular biology analysis revealed that 21-hydroxylase deficiency was found in 91.8% (n=169) of cases, while 11-β-hydroxylase deficiency was found in only 4.9% (n=9) of cases. Lastly, 3-beta-hydroxysteroid dehydrogenase deficiency in 3.2% (n=6) of cases. We analyzed the different clinical forms based on genotypic sex, biological work-up, and type of enzyme block (Table 3).

**Table 3:** Comparison between different phenotypes on the biological and molecular level.

Variables	Classical form with salt wasting (n=84)	Pure virilizing form (n=48)	The non-classical form (n=52)
8 AM cortisol level (µ/dL) (median)	4.3 (2.3-6.7)	4.5 (3.6-6.8)	18.3 (9.5-114)
Testosterone, n (%)	n=69	n=65	n=52
High	66 (95.6)	61 (93.8)	31 (59.6)
Normal	3 (4.4)	4 (6.1)	21 (40.4)
17-hydroxyprogesterone (ng/dL) (median)	9870 (3250-30080)	5560 (2400-8150)	3460 (1190-4950)
Karyotype, n (%)			
46XX	50 (34.9)	43 (30)	14 (9.8)
46XY	26 (18.2)	4 (2.8)	5 (3.5)
45X	1 (0.7)	0	0
Enzymatic block, n (%)			
21-hydroxylase	79 (42.9)	42 (22.8)	48 (26)
11-beta-hydroxylase	0	5 (2.7)	4 (2.2)
3-beta-hydroxylase	5 (2.7)	1 (0.5)	0

Glucocorticoids (hydrocortisone) were started in 93% (n=171) of children in this study, with a median dose of 15 mg/m^2^/day, combined with a mineralocorticoid in 64.1% (n=118) of cases at a median dose of 0.1 mg/day, and an anti-androgen in 28.2% (n=52) of cases. The children were closely monitored in the neonatal period and then every three months.

During monitoring, stature delay was observed in 26% (n=48), with an average of -2SDs. Obesity was observed in 4.3% (n=8), and Cushing syndrome in 2% (n=3). Precocious puberty was observed in 14% (n=26) of cases, all of whom were treated with an LH-RH analog. Testicular ultrasound revealed intra-testicular adrenal inclusions in 3.2% (n=6) of cases.

After multidisciplinary consultation, a feminizing genitoplasty was performed in 36.4% (n=67) of children in this study, with a median age of 3.25 years. While a masculinizing genitoplasty was only performed in 1% (n=2) of cases, and 3.2% (n=6) of cases benefited from a hypospadias repair. The mortality rate in this study was 3.2% (n=6) due to infectious complications.

## Discussion

CAH is a group of genetic diseases with well-defined pathophysiology and clinical consequences. Numerous studies have demonstrated a correlation between patients' phenotype and their cytogenetic profile [2]. Therefore, we conducted a study in which we retrospectively analyzed the clinical and molecular profiles of 184 patients with CAH.

In this cohort, the age of patients ranged from birth to 13 years, with a median age of 1.5 months at the time of diagnosis. These results are consistent with those reported by Suarez et al. [1]. On the other hand, Ngo et al. reported a higher median age [3].

In Morocco, CAH still poses a problem of early diagnosis due to the absence of a national neonatal screening program. In this study neonatal diagnosis was made in 33.2% (n=61) of cases. These results are lower than those reported by Suarez et al. [1]. Among them, 37.7% of cases (n=23) were boys, which can be explained by the diagnostic difficulty in boys, given the absence of external genital anomalies that can guide diagnosis.

In this study, there was a difference in the average age at diagnosis of the different clinical forms. The pure virilizing form was diagnosed earlier, with an average age of 19 days. The classical form with salt wasting was generally diagnosed between the second and third week of life [2]. However, when a screening program is implemented, we would expect an earlier age of diagnosis. The non-classical form was diagnosed at a later average age of 17 months. The sex ratio is balanced in this disease [2]. There was no sexual predominance in this study, while other studies have demonstrated a slight female predominance [1,4].

The classical form with salt wasting represents 75% of classical forms [2]. In this study, it represented 63.6% (84) of cases, which is similar to what is reported by Suarez et al. [1] and higher than the results observed in other series where it represented 36% and 33% [5,6]. These infants present a complete deficiency in mineralocorticoids and glucocorticoids, which puts their vital prognosis at risk from the first weeks of life [2]. Hypoaldosteronism causes early salt wasting leading to hypovolemia with hyponatremia and hyperkalemia resulting in severe dehydration [5]. The mean serum sodium level was 125 mEq/L and that of potassium was 6.50 mEq/L, which is similar to other studies where the mean sodium value was 124 mEq/L [1,5].

Girls present with virilization of the external genitalia at birth due to exposure to a high concentration of androgens during the critical period of genital development. Exposure to androgens during the first trimester of pregnancy results in the fusion of the labia minora and majora, with the uterovaginal canal opening into the urinary tract [2]. Subsequently, it causes abnormal growth of the clitoris, which remains sensitive to androgens throughout pregnancy and in the postnatal period. Internal genital organ development remains normal in the absence of anti-Müllerian hormone (AMH) [2]. Disorders of sexual differentiation, classified according to the Prader score, were the most frequent mode of revelation in this study, either in the context of a classical form with salt wasting or in the context of a pure virilizing form. Our results are consistent with those reported in other studies around the world [1,2].

Among the classical forms, 25% are said to be pure virilizing [2]. In these cases, 21-hydroxylase has very reduced functional activity but allows the synthesis of a sufficient amount of aldosterone to avoid symptoms related to salt wasting. The data from our series are similar to the literature since it represented 26% (n=48) of classical forms [2].

The first paraclinical examinations to be requested, in the absence of systematic neonatal screening, are a 17-OHP, anti-Müllerian hormone (AMH), and testosterone assay, as well as a pelvic ultrasound to search for Müllerian derivatives [2,7]. These examinations should help to eliminate differential diagnoses, that is to say mainly a lack of virilization in a 46XY boy or an ovotestis, by showing the existence of normal internal genital organs [2].

The diagnosis is confirmed when the 17-hydroxyprogesterone value is above 1000 ng/dL. A level between 200 ng/dL and 1000 ng/dL indicates the performance of a synacthen test [7]. A level below 200 ng/dL allows exclusion of the diagnosis [7].

In this study, the median 17-hydroxyprogesterone level was 9870 ng/dL in the classic salt-loss form, whereas, in the pure virilizing form, the mean level was 5560 ng/dL. These results are consistent with those of Suarez et al., suggesting that 17-OHP levels may predict a more severe phenotype [1].

Assigning sex in disorders of sexual development (DSDs) must be done as quickly as possible, but after multidisciplinary consent, taking into account the age at diagnosis, surgical options depending on the severity of androgenization, and the parents' wishes, which are often influenced by their sociocultural experience. The major problem arises for children born with a male morphotype, as the palpation of the gonads is not being done at birth and the child is declared a boy from the outset; it is not until a few months or years later that the parents consult for the absence of gonads [2].

In this study, among newborns declared male, 16.3% (n=30) had a Prader classification ranging from 3 to 5, a 46XX karyotype, and a pelvic ultrasound confirming the presence of female internal genital organs. Of these, 46.7% (n=14) underwent feminizing genitoplasty, while only 6.6% (n=2) opted for masculinizing genitoplasty.

According to the literature, the non-classical form is 10 times more common than the classical form, with an incidence of 1/500 to 1/1000 births [8]. However, in this study, it constituted the least frequent form. This may be because only the pediatric population was studied, and the majority of patients presenting this disorder are diagnosed in adulthood, which is consistent with a local study conducted by Zantour et al. and Draoui et al. [9,10].

Its diagnosis is often delayed because there is no systematic neonatal screening. In some cases, it is asymptomatic. The appearance of pubic hair around the age of five to seven years constitutes the first reason for consultation according to the literature [6]. Hyperandrogenism is variable, combining hirsutism, acne, fertility disorders, and advancement of bone age. Cortisol and aldosterone levels are often normal, and the risk of acute adrenal insufficiency is extremely low [6]. In this study, 19% (n=35) of cases had a stature advance of +2SD. Fourteen percent (n=26) of cases presented with early puberty, of which 19.2% (n=5) were of central origin caused by hyperandrogenism, which was present in 14% (n=26) of cases and could also be responsible for cycle disturbances, present in 11.5% (six cases), or even infertility. Our results match those of the literature [2,6].

Deficiency in 11-beta-hydroxylase accounts for 0.2-8% of CAH cases [9,11]. This deficit represented 4.8% (n=9) in this study, consistent with literature data [11,12]. Its incidence is estimated at 1/100000 live births in non-consanguineous populations and can be as high as 1/5000 in the Moroccan Jewish population [12]. It is caused by mutations in the CYP11B1 gene located on chromosome 8q21. It is diagnosed with signs of virilization of a newborn or fetus 46XX and later with hypertension, which is explained by the overproduction of deoxycorticosterone (DOC) and S compound, known by their mineralocorticoid action can generate high blood pressure. Salt-wasting syndrome is very rare [12]. In this study, arterial hypertension was observed in 22% (n=2) of cases, which is significantly lower than the study by Khelifi et al. [11]. No patient in this study had salt-wasting syndrome, which is similar to the results of Khelifi et al. [11]. A total of 55.5% (n=5) of cases presented with a pure virilizing form, and 44.4% (n=4) of cases had a non-classical form. The diagnosis is based on an elevation in the levels of DOC and S compound, with an elevation in testosterone and delta 4 androstenedione, and unlike 21-hydroxylase deficiency, reninemia is low. 17-OHP is moderately high [11].

Deficiency in 3-β-hydroxysteroid dehydrogenase (3-β-HSD) is a rare form of CAH. It is classified as the third most common after 21-hydroxylase deficiency and 11-β-hydroxylase deficiency [11]. It is responsible for 1-10% of CAH cases, consistent with the results of this study where it represented 3.3% (n=6) of cases [13]. This entity is characterized by the presence, to varying degrees, of salt-wasting syndrome and incomplete virilization of boys such as perineo-scrotal or perineal hypospadias, found in all cases in this study. A micropenis was observed in 50% (n=3) of cases (50%) and 50% (n=3) of cases had ectopic gonads. However, in girls, normal external genitalia, or virilization, at least in the form of clitoral hypertrophy, has been described [8]. They are secondary to the peripheral conversion of precursors into active androgens by the type I isoenzyme (3-β-HSDI) [13]. No girls presented with this deficit in this study.

According to the recommendations of the American Society of Endocrinology, the treatment of CAH is based on daily hydrocortisone supplementation, approximately 30 mg/m²/day in the first year of life, and then adjusted to between 10 and 15 mg/m²/day in three doses per day [7]. In this study, 93% of children were treated with a median dose of 15 mg/m²/day. Regarding mineralocorticoids, the recommended dose is 0.05-0.2 mg/day in 1-2 doses per day [7]. In this study, 64.1% (n=118) of cases were treated with a median dose of 0.1 mg/day.

The objective of treatment is to achieve satisfactory hormonal balance with minimal effective doses of hydrocortisone and fludrocortisone. Excessive doses of glucocorticoids can lead to iatrogenic Cushing's syndrome with cessation of growth and metabolic or bone complications [7]. This was observed in 1.6% (n=3) of cases in this study, which were lost to follow-up. Conversely, insufficient control of androgen secretion in children leads to pubarche or premature thelarche, or even true central precocious puberty, acne, an accelerated growth velocity with advanced bone age, and premature fusion of growth plates resulting in reduced adult height [2,5].

Controlling hyperandrogenism may require the use of androgen receptor antagonists or oral contraceptives containing drospirenone, which effectively reduce the synthesis of adrenal and ovarian androgens [8,14]. In this study, 28.2% (n=52) of cases were treated with an anti-androgen.

Surgical treatment is a cornerstone of patient care [15]. It aims to achieve a normal appearance of the external genitalia, with normal urinary tracts, without obstruction or recurrent infections, as well as a normal sexual life. In this study, feminizing genitoplasty was performed in 36.4% (n=67) of children, while masculinizing genitoplasty was only performed in 1% (n=2). A total of 3.2% (n=6) of cases benefited from a treatment for hypospadias. The age of surgery is currently a subject of controversy, with some teams advocating treatment around six months of age and others recommending it at puberty [2,15]. In this study, feminizing genitoplasty was performed in 36.4% (n=67) of the children, with a median age of 3.25 years, which is in line with the study carried out by Abosena et al. [16].

In this study, we emphasized the importance of neonatal screening for congenital adrenal hyperplasia, since the classic form was the most frequent in our series. The study had its limitations. The sample size was small, as the study was limited to a single university center, and the results cannot be generalized to a larger population. No genetic studies are available in our country. We rely essentially on molecular biology. Further multicenter longitudinal studies with large samples are recommended to support the results of this study.

## Conclusions

Congenital adrenal hyperplasia is a group of rare diseases, with 21-hydroxylase (21-OHD) deficiency the most common type of CAH (90-95%), followed by 11-β-hydroxylase block, which accounts for 8% of all CAH. In this study, the sociodemographic and clinical characteristics are similar to those reported in the literature. A total of 71.7% of cases presented a classic form while 28.3% of cases presented a non-classic form.

Disorders of sex development were the most frequent mode of presentation in this study, highlighting the importance of neonatal screening for early diagnosis. Unfortunately, it is not systematically practiced in Morocco, which makes the management of congenital adrenal hyperplasia a challenge in many fields in our country.
